# Impact of Modeling Assumptions on Stability Predictions in Reverse Total Shoulder Arthroplasty

**DOI:** 10.3389/fphys.2018.01116

**Published:** 2018-08-21

**Authors:** Mehul A. Dharia, Jeffrey E. Bischoff, David Schneider

**Affiliations:** ^1^Computational Biomechanics, Corporate Research, Zimmer Biomet, Warsaw, IN, United States; ^2^Shoulder & Elbow Institute, Panorama Orthopedics & Spine Center, Golden, CO, United States

**Keywords:** micromotion, stability, initial fixation, reverse shoulder arthroplasty, finite element analysis, screw modeling

## Abstract

Reverse total shoulder arthroplasty (rTSA) is commonly used in the shoulder replacement surgeries for the relief of pain and to restore function, in patients with grossly deficient rotator cuff. Primary instability due to glenoid loosening is one of the critical complications of rTSA; the implants are designed and implanted such that the motion between the glenoid baseplate and underlying bone is minimized to facilitate adequate primary fixation. Finite element analysis (FEA) is commonly used to simulate the test setup per ASTM F2028-14 for comparing micromotion between designs or configurations to study the pre-clinical indications for stability. The FEA results can be influenced by the underlying modeling assumptions. It is a common practice to simplify the screw shafts by modeling them as cylinders and modeling the screw-bone interface using bonded contact, to evaluate micromotion in rTSA components. The goal of this study was to evaluate the effect of three different assumptions for modeling the screw-bone interface on micromotion predictions. The credibility of these modeling assumptions was examined by comparing the micromotion rank order predicted among three different modular configurations with similar information from the literature. Eight configurations were modeled using different number of screws, glenosphere offset, and baseplate sizes. An axial compression and shear load was applied through the glenosphere and micromotion at the baseplate-bone interface was measured. Three modeling assumptions pertaining to modeling of the screw-bone interface were used and micromotion results were compared to study the effect of number of peripheral screws, eccentricities, and baseplate diameter. The relative comparison of micromotion between configurations using two versus four peripheral screws remained unchanged irrespective of the three modeling assumptions. However, the relative comparison between two inferior offsets and baseplate sizes changed depending on the modeling assumptions used for the screw-bone interface. The finding from this study challenges the generally believed hypothesis that FEA models can be used to make relative comparison of micromotion in rTSA designs as long as the same modeling assumptions are used across all models. The comparisons with previously published work matched the finding from this study in some cases, whereas the comparison was contradicting in other cases. It is essential to validate the computer modeling approach with an experiment using similar designs and methods to increase the confidence in the predictions to make design decisions.

## Introduction

**FIGURE 1:**
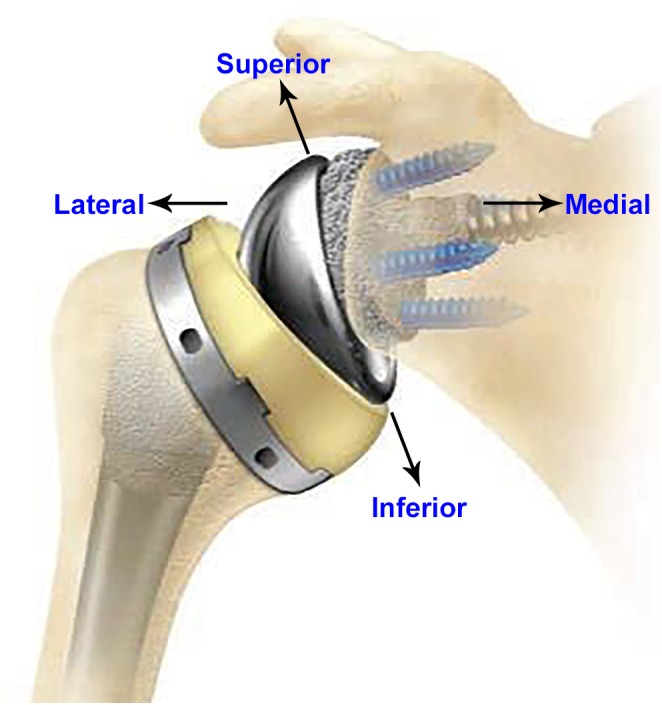
A typical rTSA system (anterior–posterior view).

Since 1972, total shoulder arthroplasty (TSA) has been used for treatment of osteoarthritis, fracture, and other shoulder non-inflammatory issues. Anatomic total shoulder arthroplasty (aTSA) and reverse total shoulder arthroplasty (rTSA) are commonly used in the shoulder replacement surgeries for the relief of pain and to restore function. Both aTSA and rTSA are considered as successful procedures with similar patient outcomes (Kiet et al., 2015). Surgeons make the decision to use aTSA or rTSA based on the condition of the joint as well as past TSA history of the patient. TSA procedures are commonly used in patients with arthritis in the joint between scapula and humeral bones, with intact rotator cuff. aTSA typically entails a stem component implanted in the humeral canal with a metal head component assembled on the superior side of the stem, articulating against a plastic socket component implanted in the scapula. Significant rotator cuff muscle damage in patients may prove inadequate to provide joint stability to the aTSA components. The component positions can be reversed in such cases (rTSA) by implanting a socket type component in the humeral bone and a metallic hemisphere component in the glenoid bone. **Figure 1** presents an example of an rTSA implant. rTSA is commonly used as a primary procedure for patients whose shoulder joint has a grossly deficient rotator cuff with severe arthropathy, and also as a revision procedure for a failed aTSA due to reduced bone stock in the humerus and scapula with deficient rotator cuff. The complication rate for rTSA has been reported as three times higher when used as revision for failed aTSA, in comparison to a primary rTSA (Boileau, 2016).

There are several complications associated with rTSA including joint instability, periprosthetic fracture, infection, and component loosening. Bohsali et al. (2017) studied complications of rTSA in 78 studies published between 2006 and 2015, including a total of 4,124 shoulders, and found that 7.2% of all complications were attributed to glenoid loosening. A literature review comprising 782 rTSA found radiographic lucent lines in the glenoid in 4.3% of the cases with postoperative problems and glenoid loosening in 5% of cases with complications (Zumstein et al., 2011). Boileau (2016) reported their experience with 825 rTSA performed between 1996 and 2013 and found the rate of glenoid loosening as a reason for reintervention at 9%.

It is essential to minimize the motion between the baseplate and the underlying glenoid bone to promote osseous integration at the bone-baseplate interface in uncemented designs and therefore achieve adequate primary fixation. However, the service loads experienced by rTSA components challenge the fixation at the interface between the bone and the baseplate. Additionally, the glenosphere can be lateralized in many systems in order to improve range of motion without impingement, which further challenges the fixation by increasing the moment arm at the bone-implant interface (Berliner et al., 2015). An increased torque of 44 and 69% at the baseplate-bone interface has been reported with increased lateral offsets of 23 and 27 mm, respectively (Harman et al., 2005). Implant designs offer a variety of mechanisms to achieve fixation stability such as various numbers of screws, screw types, screw lengths, and screw angles, as well as use of a porous material substrate to increase friction with the bone and to facilitate bony ingrowth. A narrative review cited initial baseplate screw fixation has been reported as the most important factor leading to long-term fixation through osseous integration (Berliner et al., 2015).

Micromotion between baseplate and bone is commonly used as a pre-clinical indicator for stability. ASTM F2028-14 provides a standard test method *in vitro* for evaluating glenoid loosening or disassociation by measuring displacement of the glenoid baseplate in response to axial compression and shear loading (ASTM F2028-14, 2017). This test method has been utilized to demonstrate significant differences in displacement between different screw configurations, medialized/lateralized center of rotation, and different densities of bone substrates (Roche et al., 2008, 2011; Stroud et al., 2013). Testing various configurations is complicated and time consuming. Additionally, the measurements are obtained at the edges of the baseplate or glenoid component, not representing interfacial micromotion and could be misleading (Favre et al., 2011).

Finite element analysis (FEA) simulations are often used for replicating the test setup per ASTM F2028-14, to determine worst case configuration for physical testing or to make comparisons among different designs or configurations. Several FEA studies have been performed to study the effect of implant designs, lateralization, inferior tilt of glenoid, and degree of joint conformity on glenoid baseplate-bone micromotion (Hopkins et al., 2008; Virani et al., 2008; Hopkins and Hansen, 2009; Suárez et al., 2012; Chae et al., 2016; Denard et al., 2017; Elwell et al., 2017; Geraldes et al., 2017). The complexity of the FEA models has increased with time due to improvements in computing power and software technologies. However, it is a common practice to simplify the FEA models for increasing efficiency of solution times by reducing model size. For this purpose, small features that are deemed irrelevant or are located away from an area of interest are often ignored. However, results from FEA can be critically dependent on the modeling assumptions. In particular, one commonly used simplification in rTSA modeling studies is de-featuring of the screw threads from the screw shafts and the screw holes in the bone (Hopkins et al., 2008; Virani et al., 2008; Hopkins and Hansen, 2009; Suárez et al., 2012; Chae et al., 2016; Denard et al., 2017; Elwell et al., 2017; Geraldes et al., 2017). The screw shafts are modeled as cylinders, virtually implanted in the cylindrical holes created in the bone. Further, in most cases, the interfaces between the screw shafts and the respective holes in the bone are modeled using bonded or tied contact. Similar modeling assumptions are found in studies involving modeling of screws in other joints as well (Alonso-Vázquez et al., 2004a,b). A study by Inzana et al. (2016) used a single screw-in-bone model to demonstrate that the relative comparisons of implant stability of a fracture plate in the proximal humerus remained unaffected by de-featuring the threads in the screw shaft and the holes in the bone. However, principal strains were used as a measure of stability and the measurements were in the proximity of the screw-bone interface itself. Most of the previous modeling studies have implicitly hypothesized that models can be used for one-to-one comparisons as long as the same modeling assumptions are used in all models. To the authors’ knowledge, no previous study has investigated the effects of these modeling assumptions on the resulting micromotion at the baseplate-bone interface in the rTSA components.

The aim of this study is to evaluate the impact of key modeling assumptions related to screw-bone interactions on the predicted relative motion (micromotion) between the glenoid baseplate and the bone in various rTSA configurations. Specifically, three research questions were examined to study the effect of number of screws, eccentricity, and baseplate size, on micromotion by using three different modeling assumptions to model the screw-bone interface. Further, the credibility of these modeling assumptions was examined by comparing the predicted answers for three research questions with similar information from the literature.

## Materials and Methods

### rTSA Components

**FIGURE 2:**
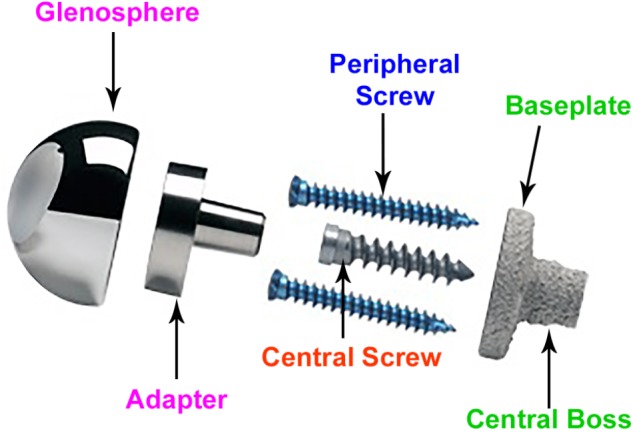
Description of rTSA components.

**Figure 2** provides the description of various components. rTSA components with two glenoid baseplate diameter sizes were selected; 25 mm and 28 mm. The underside of the baseplates has porous plasma spray (PPS^^®^^) coating to facilitate osseous integration. The baseplate designs have a short central boss housing to accommodate a central screw for rigid compression into the glenoid vault. While the central boss itself can provide resistance to shear loading, the central screw used in conjunction with the central boss can improve the compression between the baseplate and the bone to reduce micromotion at the bony interface. Additionally, the baseplate can accommodate four peripheral screws which can be inserted at variable angles. Overall, the combination of 5 screws, central boss and PPS coating offers stability for osseous integration in the months immediately following surgery. An adapter component is placed between the baseplate and the glenosphere. The adapter can be rotated to create a glenosphere offset in any direction (superior–inferior, anterior–posterior) to provide component positioning specific to patients and also avoid scapular notching while maintaining optimal range of motion.

### Study Configurations

With the modularity of implantation options described above, infinite combinations are available to the surgeons. Several combinations were selected for this study using variables in four main categories: screw-bone interface condition, number of peripheral screws, offset of the glenosphere in the superior-inferior direction, and baseplate diameter size. Configurations in all four categories are described in detail in the following sub-sections. With two combinations for number of peripheral screws, two inferior offset combinations, and two baseplate sizes, a total of 8 combinations were possible. Each of these eight combinations was studied with three different approaches for modeling the screw-bone interface. Thus, a total of 24 configurations were studied. **Table 1** presents the configurations considered in this study.

**Table 1:** Combinations considered in this study.

Combinations	Baseplate size (mm)	Number of peripheral screws	Inferior offset		Screw-bone block interface
			Type	mm	
1	28	2	Minimum	0.5	
2	28	2	Maximum	3.5	**cyl-b**
3	28	4	Minimum	0.5	OR
4	28	4	Maximum	3.5	**thr-b**
5	25	2	Minimum	1.5	OR
6	25	2	Maximum	3.5	**thr-f**
7	25	4	Minimum	1.5	
8	25	4	Maximum	3.5	

#### Screw-Bone Interface

**FIGURE 3:**
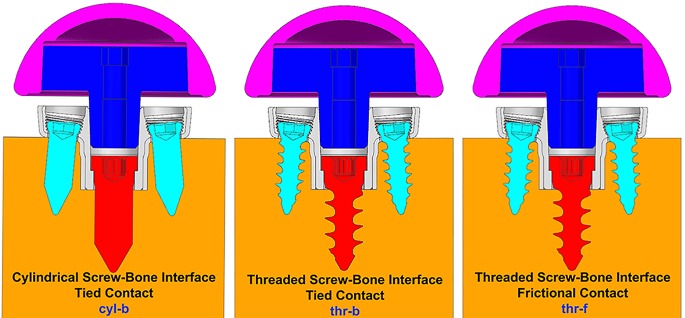
Three modeling assumptions between the screw shafts and the bone (cross-sectional view in sagittal plane).

Three configurations were considered representing different ways to model the interface between screws and the bone. (1) Screw shafts were modeled as cylinders without threads. The interface between screws and the bone was modeled using rigidly bonded contact. This configuration will be referred to as **cyl-b**. The outer thread diameter was used to model the cylinders representing screw shafts. (2) Screw threads were modeled on the screw shafts and corresponding thread geometries were modeled in the bone. The screw-bone interface was modeled using rigidly bonded contact. This configuration will be referred to as **thr-b**. (3) Similar to configuration 2, but the screw-bone interface was modeled using sliding friction with coefficient of friction 0.3 (Inzana et al., 2016). This configuration will be referred to as **thr-f**. **Figure 3** illustrates the three modeling assumptions between the screw shafts and the bone.

**FIGURE 4:**
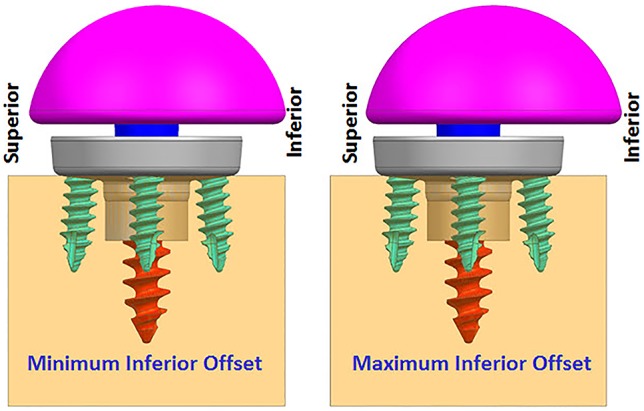
Finite element analysis (FEA) models with two offset conditions (anterior–posterior view).

#### Number of Peripheral Screws

As described before, this implant allows the surgeon to use up to four peripheral screws based on the quality of the bone and the amount of fixation deemed necessary for a specific patient. In this study, two of many possible combinations were selected. (1) Two peripheral screws; one each on the inferior and superior sides. (2) Four peripheral screws; one each on the inferior and superior sides, and one each on the anterior and posterior sides.

#### Eccentricity

While infinite combinations of eccentricities in anterior-posterior and inferior–superior directions are available by allowable glenosphere rotations through use of an adapter, two combinations of offsets in inferior-superior directions were selected, (1) Minimum inferior superior offset and (2) Maximum inferior offset. A minimum offset of 0.5 mm and 1.5 mm was used for the 28 mm and 25 mm baseplate, respectively, based on the glenosphere compatibility. For both baseplates, a maximum offset of 3.5 mm was used.

#### Baseplate Size

Two baseplate diameter sizes were considered, (1) 25 mm and (2) 28 mm, combined with a size 36 and 41 mm glenosphere, respectively.

### Research Questions

This study performed FEA on the above described configurations to answer three questions pertaining to micromotion at the interface between the glenoid baseplate and the bone.

(1) Which number of peripheral screws (2 or 4) results in lower micromotion?
(2) Which eccentricity in inferior-superior direction (minimum inferior superior offset or maximum inferior offset) results in lower micromotion?
(3) Which baseplate size (25 or 28 mm) results in lower micromotion?

Finally, the main question was whether the approach for modeling the screw-bone interface affects the answers to the above three questions.

### Test Method

The experimental setup, per ASTM F2028-14, measures the initial glenoid baseplate fixation to the bone before cyclic loading. Fixation is measured as an axial load of 430N is applied approximately through the center of rotation, perpendicular to the glenoid plane, and as a shear load of 350N is applied parallel to the glenoid plane. The resulting displacement of the baseplate in the direction of applied axial compression and shear loads is measured as a fixation response. Then, the glenoid components are rotated about the humeral liner for a fixed number of cycles as an axial compressive load of 750N is applied through the humeral liner to the glenoid component, to load the assembly in a physiologically relevant manner. After the cyclic loading is completed, glenoid baseplate fixation to the bone is measured again in both directions as described previously.

**FIGURE 5:**
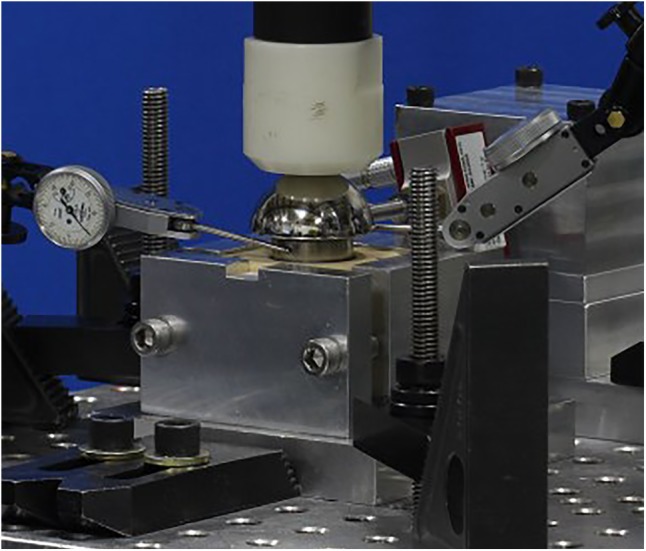
A representative laboratory test setup showing axial compression and shear load cells.

Finite element analysis models were created to simulate the experimental setup described above without considering the cyclic loading aspect. rTSA glenoid components were virtually implanted using the screw solids into a block of material representing the idealized bone, as prescribed by the surgical technique. A load of 756N, representing one body weight, was applied through the glenosphere at the center of rotation in axial compression direction perpendicular to the glenoid baseplate. An additional load of 756N was also applied in the shear direction, parallel to the glenoid baseplate plane, creating a resultant load of 1070N. This load magnitude was derived in a previous study as high-impact daily loading by reviewing a series of shoulder motion analyses (Anglin and Wyss, 2000), and is more rigorous than that prescribed by ASTM F2028-14. This technique of applying axial compression and shear load creates eccentric loading conditions similar to the rocking-horse loosening mechanism and is consistent with what has been used in previously reported physical testing as well as FEA studies (Anglin et al., 2000; Harman et al., 2005; Hopkins et al., 2008; Mroczkowski, 2008; Virani et al., 2008; Hopkins and Hansen, 2009; Bergmann et al., 2011; Chae et al., 2016) to evaluate micromotion at the interface between the bone and the baseplate. **Figure 5** presents a representative laboratory test setup replicated by the FEA models.

### Finite Element Model

#### System Configuration

Finite element analysis models included components described in Section “rTSA Components.” Three dimensional computer aided design models were created at nominal dimensions using NX version 8.5 (Siemens PLM Software, Plano, TX, United States). All FEA models were created by importing the CAD models into ANSYS (Workbench 16.2, Ansys, Inc., Canonsburg, PA, United States).

#### System Properties

All the components were modeled using linear elastic material properties. It was assumed that deformation of the components is small under this test condition and therefore, modeling the non-linear material response of the system was not deemed necessary. For all models, the predicted stress response of the critical components was below the yield strength, justifying this assumption.

The baseplate, adapter, and screws were modeled using Titanium Ti-6Al-4V material (*E* = 1.1e5 MPa, nu = 0.3) (Boampong et al., 2003). The glenosphere was modeled using Cobalt-Chromium-Molybdenum material properties (*E* = 2.2e5MPa, nu = 0.3) (ASTM F75, 2000). The solid polyurethane foam used as a bone substitute is not intended to replicate the mechanical properties of the human bone, however, it does provide consistent and uniform material with properties in the range of human cancellous bone. The bone block was modeled using bone foam properties (*E* = 193 MPa, nu = 0.3) (ASTM F1839-08, 2016).

#### System Conditions

##### Loading and support conditions

A small patch was defined at the center of the proximal glenosphere dome to apply load. An axial compression load of 756 N was applied through that load patch in the direction perpendicular to the baseplate. An additional 756 N load was applied in the shear direction through the same load patch, parallel to the baseplate. The distal surface and the side faces of the rectangular bone block were constrained against motion in all degrees of freedom.

##### Interactions

The central and peripheral screw heads were connected to the baseplate holes using tied contact. The connection between the glenosphere and the adapter, as well as the connection between the adapter and the baseplate was modeled using tied or bonded contact. The contact between the baseplate and PPS coating was also modeled using bonded contact to capture the PPS coating bonded to the baseplate. The interface between the PPS coating with the bone block was modeled using a coefficient of friction of 0.64 (Biemond et al., 2011). The interface between screws and bone block were modeled as described in Section “Screw-Bone Interface.”

#### System Discretization

All components were meshed using 10-node tetrahedron elements with consistent mesh density among specific components in all the models. To ensure that the results were not affected by the mesh size selection, a mesh refinement study was performed upfront using two models; one each with two and four peripheral screws; configurations 1 and 3 in **Table 1**. Starting with 1mm mesh size, the mesh at the interface between baseplate and the bone was refined by cutting the mesh size into half until the relative difference in micromotion predictions between iterations was within 5% of each other, an acceptable threshold for comparative device evaluations (ASTM F2996-13, 2013). Using a mesh size of 0.5 mm at the baseplate-bone interface, the % difference in predicted peak micromotion was within 5 and 3% for the configurations 1 and 3, respectively, compared to the respective models using 1 mm mesh size. With further mesh refinement using 0.25 mm mesh size at the baseplate-bone interface, the predicted peak micromotion was within 3% of the previous iteration for both configurations. Therefore, the mesh size of 0.5 mm at the baseplate-bone interface was considered as the converged mesh size and was used for all the other configurations in **Table 1**. Thus, micromotion results in any two models which are within 5% of each other were considered equal.

#### Numerical Implementation

All analyses were performed using ANSYS version 16.2 FEA software. Non-linear static analyses were performed using an implicit solver. The default convergence criteria and iteration methods were used. FEA were performed in two steps. In the first step, 750N axial compression load was applied, followed by the 750N shear load application in the second step.

#### Results Postprocessing

The peak micromotion predicted at the interface between the baseplate and the bone was recorded for all the configurations. The relative motion of the baseplate with the bone includes two tangential components [inferior–superior (IS) and anterior–posterior (AP)], as well as the normal medial-lateral (ML) component. Most experimental implementations of F2028-14 measure only selected components of the micromotion, and also only at the outside edge of the baseplate. Thus, the overall micromotion across the baseplate-bone interface may be poorly characterized. FEA models can provide greater insight into the micromotion response by quantifying all components of micromotion across the entire interface. The relative displacement between glenoid baseplate and the interfacing bone block was computed as a vector composition of the tangential and normal micromotion (Viceconti et al., 2006; Favre and Henderson, 2016).

## Results

**FIGURE 6:**
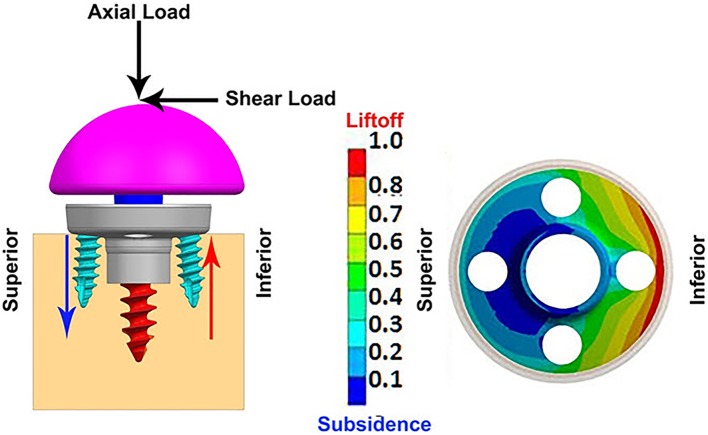
**(Left)** FEA model of 25 mm baseplate implanted using 2 peripheral screws (view in sagittal plane); red and blue arrows showing resulting rocking motion. **(Right)** Interfacial normalized micromotion plot in distal view (results shown for configuration using thr-f screw-bone interface).

**Table 2** presents the micromotion results predicted for all eight combinations, using all three screw-bone modeling assumptions. Among all 24 combinations, the highest micromotion value was predicted for the combination that used the 25 mm baseplate implanted with two peripheral screws and 1.5 mm inferior offset. **Figure 6** presents the model setup resulting in rocking motion of the baseplate with respect to the bone and resulting interfacial micromotion plot for this configuration. Micromotion results for all the combinations were normalized against this highest micromotion value.

**Table 2:** Normalized micromotion results.

Combinations	Baseplate size (mm)	Number of peripheral screws	Inferior offset		Screw-bone block micromotion		
			Type	mm	cyl-b	thr-b	thr-f
1	28	2	Minimum	0.5	0.698	0.665	0.890
2	28	2	Maximum	3.5	0.628	0.564	0.793
3	28	4	Minimum	0.5	0.625	0.582	0.826
4	28	4	Maximum	3.5	0.549	0.512	0.738
5	25	2	Minimum	1.5	0.570	0.677	1.000
6	25	2	Maximum	3.5	0.652	0.616	0.902
7	25	4	Minimum	1.5	0.454	0.564	0.866
8	25	4	Maximum	3.5	0.558	0.509	0.823

### Overall Micromotion

The overall peak micromotion at the baseplate-bone interface was contributed by the relative motion in both the tangential (superior–inferior) as well as normal (medial-lateral) directions. The peak micromotion was located toward the inferior edge. On average, the contribution by inferior-superior tangential motion was 20–30% higher than that of the normal motion at the inferior edge. For all eight combinations, models using thr-f screws-bone interface predicted significantly higher micromotion; 26–91% higher compared to models using cyl-b interface, 34–62% higher compared to thr-b interface. Among models using bonded contact at the screw-baseplate interface, models using cyl-b interface predicted marginally higher micromotion (5–11% higher) compared to those using thr-b interface. However, for two models using 25 mm baseplates with minimum inferior offset, this trend was reversed, resulting in 16–19% decreased micromotion in models using cyl-b interface with 2 and 4 peripheral screws, respectively. Following paragraphs evaluate the three specific Research questions listed in Section “Eccentricity.”

### Number of Peripheral Screws

**FIGURE 7:**
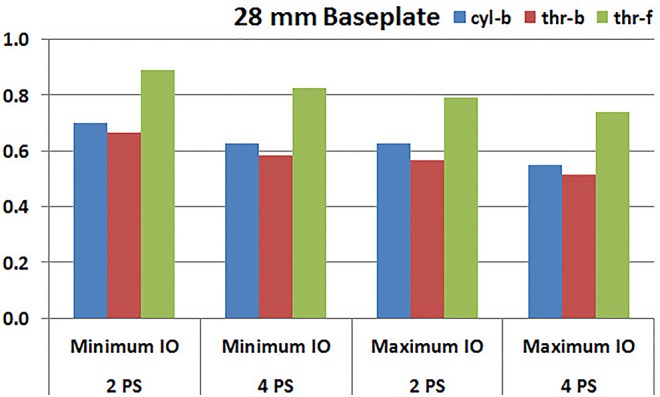
Interfacial micromotion results of 28 mm diameter baseplate using two and four peripheral screws (2PS and 4PS); Minimum and Maximum inferior offset (Minimum IO and Maximum IO).

**FIGURE 8:**
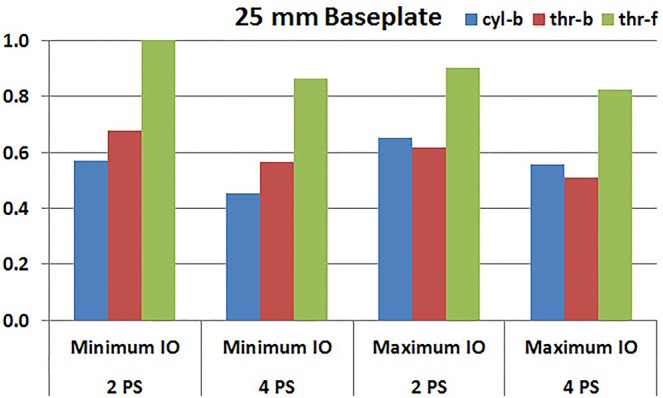
Interfacial micromotion results of 25 mm diameter baseplate using two and four peripheral screws (2PS and 4PS); Minimum and Maximum inferior offset (Minimum IO and Maximum IO).

**Figures 7**, **8** plot the interfacial micromotion results showing the effect of implanting different number of peripheral screws on predicted micromotion using 28 and 25 mm diameter baseplates, respectively. Irrespective of the screw-bone interface used, the combination using four peripheral screws resulted in less micromotion than the respective combination using two peripheral screws. The rates of reduction were less pronounced when thr-f interface was used, compared to cyl-b (31–45% lower) and thr-b (20–49% lower) interfaces.

### Eccentricity

**FIGURE 9:**
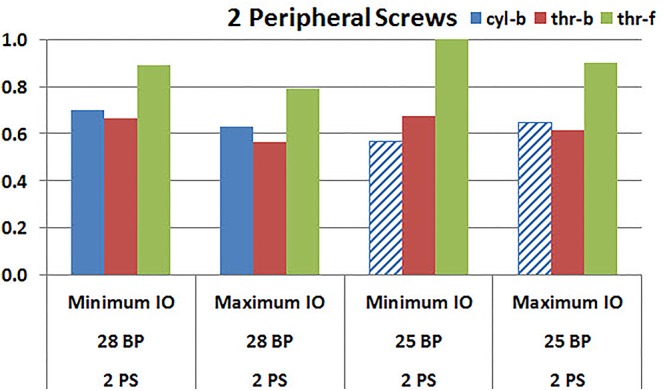
Interfacial micromotion results using Minimum and Maximum inferior offset (Minimum IO and Maximum IO); and 28 mm and 25 mm baseplates (28 BP and 25 BP), with two peripheral screws. *The patterned bars represent the predictions where the primary trend (maximum inferior offset resulted in reduced micromotion) was reversed.*

**FIGURE 10:**
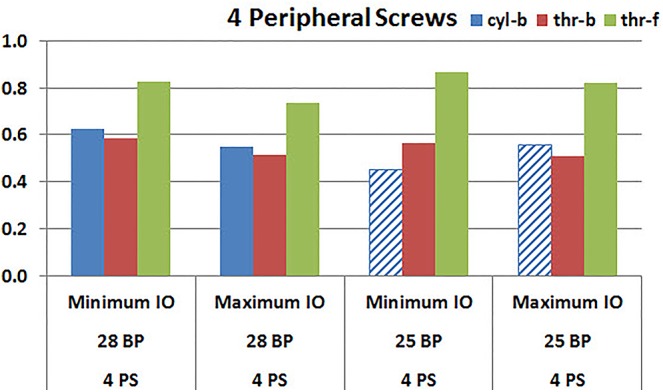
Interfacial micromotion results using Minimum and Maximum inferior offset (Minimum IO and Maximum IO); 28 mm and 25 mm baseplates (28 BP and 25 BP), with four peripheral screws. *The patterned bars represent the predictions where the primary trend (maximum inferior offset resulted in reduced micromotion) was reversed.*

**Figures 9**, **10** plot the micromotion results showing the effect of implanting two different offsets in superior-inferior direction on predicted micromotion using 2 and 4 peripheral screws, respectively. For all three screw-bone interface assumptions, maximum inferior offset resulted in lower micromotion than the minimum inferior offset in models using 28 mm baseplate. This trend was independent of the number of peripheral screws implanted. While the same trend was mostly predicted in models using 25 mm baseplate as well, the trend was reversed when screw-bone interface was modeled using cyl-b configuration; where 14 and 23% increase in micromotion was demonstrated using 2 and 4 peripheral screws, respectively. The patterned bars in the **Figures 9**, **10** represent the predictions where the primary trend (maximum inferior offset resulted in reduced micromotion) was reversed.

### Baseplate Size

**FIGURE 11:**
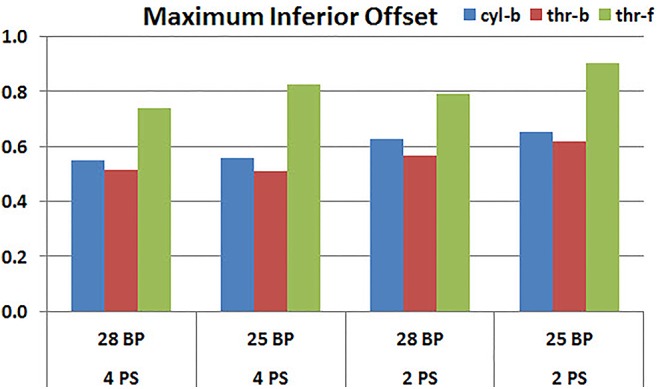
Interfacial micromotion results using 28 and 25 mm baseplates (28 BP and 25 BP) and four and two peripheral screws (4PS and 2PS), with maximum inferior offset.

**FIGURE 12:**
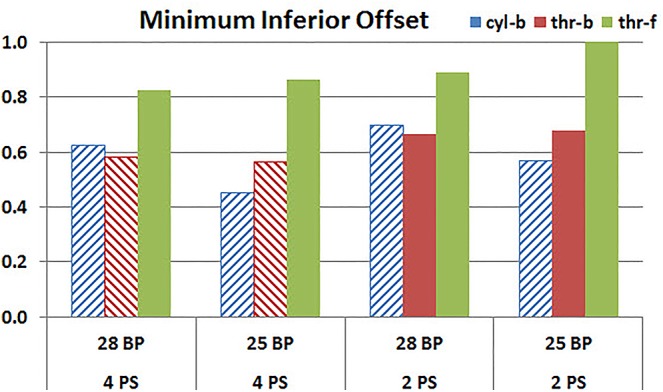
Interfacial micromotion results using 28 and 25 mm baseplates (28 BP and 25 BP) and four and two peripheral screws (4PS and 2PS), with minimum inferior offset. *The patterned bars represent the predictions where the primary trend (smaller baseplate resulted in increased micromotion) was reversed.*

**Figures 11**, **12** plot the micromotion results showing the effect of baseplate size on micromotion using maximum and minimum inferior offset, respectively. Models with the larger 28 mm baseplate resulted in lower micromotion than the smaller 25 mm baseplate, when implanted with maximum inferior offset, irrespective of the screw-bone interface assumption. However, when implanted using minimum inferior offset with 4 peripheral screws, this trend was reversed for the cyl-b and thr-b modeling assumptions at the screw-bone interface. A similar trend reversal was also shown for cyl-b modeling assumption for the minimum inferior offset implanted with 2 peripheral screws. The patterned bars in the **Figure 12** represent the predictions where the primary trend (smaller baseplate resulted in increased micromotion) was reversed.

## Discussion

The objective of this study was to evaluate whether the method in which the screw-bone interface is modeled within FEA studies of rTSA baseplate stability affects the resulting micromotion predictions, by answering three specific research questions relevant to implant design and intra-operative decision making:

(1) Does the use of more peripheral screws increase the stability of the baseplate?
(2) Does increased inferior eccentricity of components result in increased or decreased baseplate micromotion?
(3) Is a smaller or larger baseplate more stable?

This was examined by looking at model predictions for two and four peripheral screws; two differing amounts of inferior offset; and two different baseplate sizes.

Configurations with two peripheral screws resulted in higher predicted micromotion than the respective configurations using four peripheral screws, for all three methods of modeling the screw-bone interface; the modeling assumption used to model the screw-bone interface had no impact on these predictions. This consistency was not seen, however, when addressing baseplate eccentricity (superior-inferior offset) and baseplate size. Specifically, for the smaller baseplate diameter and four peripheral screws (**Figure 10**), the defeatured screw-bone interface resulted in an increase of micromotion with maximum inferior offset. For those same configurations, when using the fully featured screw geometry (with bonded or frictional contact), maximum inferior offset led to decreased micromotion. Similarly, for the minimum inferior offset configurations, the impact of baseplate size on predicted micromotion was dependent on the screw-bone interface model. As a specific example, when using four peripheral screws and a minimum inferior offset (**Figure 12**), higher micromotion was predicted for the 28 mm baseplate than for the 25 mm baseplate when the screw was bonded to the bone (whether or not the threads were explicitly modeled); whereas the opposite trend was predicted when using fully featured screws with frictional contact.

Out of the three research questions considered in the present study, therefore, the answer to only one question (do more peripheral screws result in increased stability?) was insensitive to how the screw-bone interface was modeled. For the other two questions, however, for selected configurations (e.g., the specific number of peripheral screws, inferior offset, and baseplate diameter), the predicted basic trends were *reversed* based on the way in which the screw-bone interface was modeled. This directly highlights the criticality of the modeling assumptions, even for a direct relative comparison across reasonably comparable system configurations. Further, it motivates careful scrutiny based on the *mechanical basis* of the findings from these modeling approaches, in order to ensure that predicted trending is a reasonable representation of reality.

### Mechanical Basis

For each of the eight configurations (in terms of the number of peripheral screws, inferior offset, and baseplate diameter), increased micromotion was consistently predicted when using fully featured screws in frictional contact with the bone (thr-f). This is attributed to the increased compliance of the system that is associated with frictional contact, rather than bonded contact. Further, for most configurations, minimum micromotion was predicted when using fully featured screws with bonded contact (thr-b), as compared to defeatured screws with bonded contact (cyl-b). This may be attributed to fully featured screws having a larger area between the screw and the bone in bonded contact, and therefore adding rigidity to the system as compared to the defeatured (cylindrical) screws. However, this trend was not consistent across all configurations. Further, the extent to which this trend would be impacted by modeling the cylinders using the average thread diameter, or the inner thread diameter, as opposed to the outer thread diameter as used in the current study, is unknown.

At this point, the limit to effectively judge the credibility of modeling assumptions has been reached regarding the extent to which model predictions can be trusted to draw conclusions on device performance. To ensure that correct trending is predicted requires *model validation*, even for evaluating relative performance.

### Model Validation

Various experimental studies documented in the literature have set out to address the influence of peripheral screws, eccentricity, and baseplate size on micromotion in rTSA (Humphrey et al., 2008; Hoenig et al., 2010; Poon et al., 2010; James et al., 2013; Chae et al., 2014; Formaini et al., 2017), and potentially could be used to deduce which, if any, of the three approaches considered here for modeling the screw-bone interface is accurate.

#### Peripheral Screws

Hoenig et al. (2010) reported a decrease in micromotion with the use of a higher number of peripheral screws, using loading conditions that closely match the current simulation study, and thus seemingly reinforcing the conclusions here. However, James et al. (2013) showed no significant impact of the number of peripheral screws on micromotion. This latter study used a different combination of loading than the current simulation study, as well as different metrics for assessing micromotion and a different base implant design. Further, the authors hypothesized that the lack of measurable difference in motion between two and four peripheral screws in their study may be due to variations in reaming of the glenoid due to differences in bone erosion and initial drill location, as well as due to variations in impaction of the baseplate which could permit a gap between the baseplate and the bone, none of which were incorporated in the current study.

#### Eccentricity

While a glenosphere with inferior eccentricity has been shown to have good clinical outcomes (De Biase et al., 2013) mainly due to improvement in glenoid notching, an increased baseplate micromotion has been reported in a dynamic *in vitro* test (Poon et al., 2010), which is contrary to the finding from the current study. While the increase in micromotion reported by Poon et al. (2010) was small, their study also had other variables such as design differences, lateral offsets which could have contributed to the micromotion and the difference in micromotion between each design cannot be attributed to the eccentricity alone. The micromotion measurement itself was obtained by measuring differences in motion between one point on glenosphere and three points on the glenoid bone; which could be misleading the micromotion assessment compared to recommendations for accurate interface micromotion (Favre et al., 2011) used in this FEA study. The micromotion measurement in the *in vitro* test seem to have measured tangential motion, whereas the predictions from FEA study also included the motion in medial-lateral (lift-off) direction, which was one of the main contributor to the overall peak micromotion at the inferior edge of the baseplate. Further, the FEA setup in current study has the load vector located laterally on the glenoid, which uses a shear load vector directed from inferior to superior direction along with axial compression load, creating a resultant load vector that is passing through the baseplate more inferiorly with maximum inferior offset than minimum inferior offset, which explains the reduced micromotion prediction at the inferior edges of the baseplate with maximum inferior offset. The loading used in the mechanical testing study (Poon et al., 2010) used cyclic loading and the orientation of the eccentricity used may not be matching the one used in this study.

#### Baseplate Diameter

A previous study used biomechanical testing on cadaveric specimens to study the effect of baseplate size on micromotion at the baseplate-bone interface (Chae et al., 2014). In that study, a larger 29 mm baseplate size resulted in larger surface area at the micromotion site at the baseplate-bone interface than the smaller 25 mm baseplate, resulting in an increased micromotion. This finding is contrary to the determination in the current FEA study for the maximum inferior offset configurations, as well as to the findings of another study by Hopkins and Hansen (2009) which suggested that a larger implant surface area should provide a greater resistance to interfacial micromotion than a smaller implant. However, in the biomechanical testing study (Chae et al., 2014), due to insufficient bone stock of the small glenoid for the fixation of the larger 29 mm baseplate, shorter length peripheral screws were used than the ones used to fix the 25 mm baseplate, which could potentially influence the increase in micromotion in the larger 29 mm baseplate. In the present study, higher micromotion was predicted in the smaller size baseplate for some configurations with minimum inferior offset using cyl-b and thr-b modeling assumptions, matching the findings of the biomechanical study.

The discussion here follows a common practice found in the modeling literature, namely to speak to the validity of the model using experimental data from independent laboratories. However, as seen above, rarely are such studies conducted using equivalent test conditions, in terms of system geometries, loading parameters, etc. When there are differences between model and test setup, it is then difficult to judge whether apparently complementary or apparently conflicting test results are most appropriate for assessing the validity of the model (Pathmanathan et al., 2017). It was beyond the scope of this study to formally validate this modeling approach either by comparing to the literature studies or by conducting an independent validation study. As such, it is not possible here to state unequivocally which of the three modeling assumptions used in this study gives the correct answer. Rather, the goal was to highlight the importance of basic modeling assumptions on basic modeling results; and provide guidance on key points that, if followed, can improve the utilization of computational models for rTSA stability.

### Significance

The results from this study highlight two key points that can guide the development, utilization, and interpretation of modeling studies to evaluate micromotion performance in rTSA.

#### Quantitative Device Performance

One trend consistent in all the configurations examined here is that modeling of fully featured screws, with frictional contact between the screw and the bone, results in the largest quantitative predictions of baseplate micromotion. This is the most expensive simulation considered here; and is the one that consistently resulted in the highest predictions of micromotion. It is thus an important finding for researchers who may want to employ FEA studies to calculate absolute values of micromotion for a given design or configuration, in order to make a design safety decision relative to an acceptable threshold of micromotion conducive for primary stability. Reducing the complexity of the screw-bone interface (cyl-b and thr-b) results in lower predicted values for micromotion, and therefore may lead to a false sense of confidence in the stability of the device.

#### Direct Model Validation

When comparing different configurations within a single device family, as was done here, basic performance questions were directly and meaningfully impacted by the method in which the screw-bone interface was modeled. This would be exacerbated further when comparing across different devices, in which additional parameters held constant here (frictional coefficient; effective screw diameter; etc) would vary across the models. Experimental studies documented in the literature, in many cases, are poor surrogates for direct model validation; the level of agreement, whether finally judged to be adequate or inadequate based on the purposes of the study, may be a consequence of good modeling fortune rather than engineering insight. Rigorous hierarchical model validation may not always be required; but careful consideration to model validity, even in cases of comparing across configurations, is warranted.

### Limitations

There are several limitations in this study. The actual screw preloads were not incorporated in the model, which would compress the baseplate against the bone, potentially reducing the micromotion. The findings were not confirmed with additional sensitivity studies such as different load vector orientations, screw diameters, screw lengths, bone material properties, coefficient of friction at the micromotion interface etc; many of which have been shown to have an impact on micromotion at the implant-bone interface (Hopkins et al., 2008; Hopkins and Hansen, 2009; Favre et al., 2011). The axial compression and shear loading was applied in two sequential steps. If both the loads were simultaneously applied, its effect on micromotion could have been different. The de-featured screw shafts in the configurations using cyl-b screw-bone modeling assumptions were modeled using outside thread diameters. The effect of modeling them at inner or average thread diameter was not studied. While an infinite number of combinations are possible for implantation due to modularity of various components, only eight combinations were explored in this study to investigate the impact of three modeling assumptions. However, the differences in results have already shown that the modeling assumption can impact the relative comparisons among these eight combinations.

Finally, this FEA modeling approach has not been validated against an appropriate test or set of tests, as the appropriate model validation was outside the scope of this study. While it may not be pragmatic to conduct a large number of physical tests for the purposes of model validation, a subset of the configurations analyzed here could be directly tested to ensure consistency between the parametric sensitivities predicted here and those measured experimentally. Such validation experiments likely would necessitate use of alternative micromotion metrics, as the full-field interfacial micromotions predicted here generally cannot be accessed experimentally. With such data, the appropriate set of modeling assumptions could be identified, or additional variations of the existing model (including further sensitivities identified in the preceding paragraph) could be implemented to achieve the desired accuracy.

## Conclusion

Three levels of model fidelity were tested for modeling the screw-bone interface with increased modeling complexity, model size and solution time, differentiated by whether the screw thread was explicitly geometrically modeled and by whether the screw was bonded to the bone. Using each modeling assumption, three specific questions of interest were examined within a single baseplate system based on the relative comparison of implant-bone micromotion predictions. The rank order among eight different configurations was not impacted for one of those three questions of interest; effect of number of screws. However the modeling assumptions resulted in a different rank order among eight configurations for the other two questions; effect of eccentricity in superior-inferior direction and baseplate size. If a design safety decision regarding primary stability is made solely based on absolute values of micromotion predicted by computational models, modeling simplifications at the screw-bone interface may result in lower micromotion values, and therefore may lead to an incorrect decision. Moreover, these findings demonstrate the importance of carefully evaluating the underlying modeling assumptions used to evaluate differential performance of interfacial micromotion between different rTSA configurations and designs. It further promotes an argument for performing sufficient model validation even for comparative analyses.

## Author Contributions

MD and JB conceived of the presented idea. MD carried out the implementation including developing FEA models, performing simulations, interpreting the results, and working on the manuscript. JB verified the analytical methods, results interpretation, and worked on the manuscript. DS encouraged MD and JB to investigate the topic and provided critical feedback from a practicing surgeon’s viewpoint. All authors discussed the results and contributed to the final manuscript.

## Conflict of Interest Statement

MDand JB are paid employees of Zimmer Biomet. DS is a paid consultant of Zimmer Biomet. The reviewer AB and handling Editor declared their shared affiliation at time of review.
